# *PtrWOX13A* Promotes Wood Formation and Bioactive Gibberellins Biosynthesis in *Populus trichocarpa*

**DOI:** 10.3389/fpls.2022.835035

**Published:** 2022-06-28

**Authors:** Yang Zhang, Yingying Liu, Xueying Wang, Ruiqi Wang, Xuebing Chen, Shuang Wang, Hairong Wei, Zhigang Wei

**Affiliations:** ^1^Engineering Research Center of Agricultural Microbiology Technology, Ministry of Education, Heilongjiang University, Harbin, China; ^2^Heilongjiang Provincial Key Laboratory of Plant Genetic Engineering and Biological Fermentation Engineering for Cold Region, School of Life Sciences, Heilongjiang University, Harbin, China; ^3^State Key Laboratory of Tree Genetics and Breeding, Northeast Forestry University, Harbin, China; ^4^College of Forest Resources and Environmental Science, Michigan Technological University, Houghton, MI, United States

**Keywords:** *PtrWOX13A*, wood formation, bioactive gibberellins biosynthesis, *Populus trichocarpa*, coordinated regulation

## Abstract

WUSCHEL-related homeobox (WOX) genes are plant-specific transcription factors (TFs) involved in multiple processes of plant development. However, there have hitherto no studies on the WOX TFs involved in secondary cell wall (SCW) formation been reported. In this study, we identified a *Populus trichocarpa* WOX gene, *PtrWOX13A*, which was predominantly expressed in SCW, and then characterized its functions through generating *PtrWOX13A* overexpression poplar transgenic lines; these lines exhibited not only significantly enhanced growth potential, but also remarkably increased SCW thicknesses, fiber lengths, and lignin and hemicellulose contents. However, no obvious change in cellulose content was observed. We revealed that *PtrWOX13A* directly activated its target genes through binding to two *cis*-elements, ATTGATTG and TTAATSS, in their promoter regions. The fact that *PtrWOX13A* responded to the exogenous GAs implies that it is responsive to GA homeostasis caused by GA inactivation and activation genes (e.g., *PtrGA20ox4*, *PtrGA2ox1*, and *PtrGA3ox1*), which were regulated by PtrWOX13A directly or indirectly. Since the master switch gene of SCW formation, *PtrWND6A*, and lignin biosynthesis regulator, MYB28, significantly increased in *PtrWOX13A* transgenic lines, we proposed that *PtrWOX13A*, as a higher hierarchy TF, participated in SCW formation through controlling the genes that are components of the known hierarchical transcription regulation network of poplar SCW formation, and simultaneously triggering a gibberellin-mediated signaling cascade. The discovery of *PtrWOX13A* predominantly expressed in SCW and its regulatory functions in the poplar wood formation has important implications for improving the wood quality of trees *via* genetic engineering.

## Introduction

WUSCHEL-related homeobox (WOX) family, a plant-specific clade of homeobox transcription factors (TFs) (Park et al., 2005), have been found to play an important role not only in plant developmental tissues and organs (Cheng et al., 2018; Hao et al., 2019) but also in plant response to abiotic stresses (Yang et al., 2017; Minh-Thu et al., 2018; Sajjad et al., 2021). The WOX proteins usually have a highly conserved homeodomain harboring a short stretch of amino acids (60–66 residues) with a helix–loop–helix–turn–helix structure (Yang et al., 2017), which is responsible for binding to a specific DNA sequence (Alvarez et al., 2018; Minh-Thu et al., 2018). To date, *WOX* genes have been identified in various plant species at the whole genome scale (Deveaux et al., 2008; Zhang et al., 2010; Gambino et al., 2011; Cao et al., 2017; Chang et al., 2020; Daude et al., 2020; Tang et al., 2020). The numbers of *WOX* genes in plants have been reported to be more related to the levels of ploidy and duplication events rather than genome sizes (Li et al., 2019; Wang et al., 2019; Wu et al., 2019). For example, although *Brassica napus* and *Phyllostachys edulis* both are tetraploid genome, *B. napus* has the largest *WOX* family consisting of 52 members (Li et al., 2019), while the *WOX* family *P. edulis* only has five members (Xu et al., 2019). The WOX genes have been clustered into three clades, known as WUS/modern clade, intermediate clade, and ancient clade (van der Graaff et al., 2009). For example, *AtWOX1-7* and *AtWUS* of *Arabidopsis thaliana* belong to the WUS/modern clade, *AtWOX8*-9 and *AtWOX11-12* belong to the intermediate clade, and *AtWOX10* and *AtWOX13-14* are of the ancient clade (Sarkar et al., 2007; Stahl et al., 2009). In addition, each clade of the *WOX* family has been found to contain specific conserved motifs (Kieffer et al., 2006; Deveaux et al., 2008; Ikeda et al., 2009), such as ancient clade having NVFYWFQNH motif and an ancient/basal motif (Wu et al., 2019).

It has been reported that *WOX* genes participate in multiple developmental processes in plants (Rathour et al., 2020; Han et al., 2021), such as stem cell maintenance in the shoot and root apical meristems, cambium and embryo apical–basal patterning, and lateral organ formation (Deveaux et al., 2008; Stahl et al., 2009; Ji et al., 2010; Zhang et al., 2010; Suer et al., 2011). Besides these, mounting evidence suggests that some members of the *WOX* ancient clade play a function in wood formation. For example, *AtWOX13* has been reported to be expressed not only in meristematic tissues but also in replums, lateral roots, and root vasculature (Romera-Branchat et al., 2013). *AtWOX14* overexpression induces radial growth with strong lignification of vascular stem tissues (Denis et al., 2017). *GhWOX13* participates in cotton fiber elongation (He et al., 2019). In addition, there is evidence of the link between *WOXs’* function and the gibberellin (GA) biosynthesis pathway. For example, DWT1, which encodes a WOX TF homologous to the *Arabidopsis* WOX8 and WOX9, regulates the internode elongation which is directly or indirectly associated with GA signaling (Wang et al., 2014). The expression levels of *OsWOX12A* and *OsWOX12B* were greatly upregulated by GAs after a 3-h treatment (Cheng et al., 2014). *WOX14* overexpression upregulated *GA3ox* while repressing *GA2ox*, resulting in the accumulation of bioactive GA (Denis et al., 2017). However, up to now, there is a lack of reports regarding the involvement of WOX ancient clade members in the wood formation and GA homeostasis in woody plants such as trees, especially the exact roles they play during secondary cell wall (SCW) formation.

Being the most abundant biomass produced by land plants, wood, mainly comprising dead SCW, serves as raw material for multiple end uses by humans, and is considered to be one of the most environmentally cost-effective and renewable sources of bioenergy (Zhong et al., 2010). To genetically modify wood properties, it is essential to unveil the regulatory genes that control the various developmental processes of SCW in woody plants, especially in trees. Past studies have revealed that SCW biosynthesis is mainly controlled by a hierarchical transcription regulatory network, where the high-hierarchical regulators or switches activate the middle-level master hubs and then the lower-level *TFs*, and they together coordinately regulate the expression of bottom structural genes responsible for different SCW component biosynthesis (Zhou et al., 2009; Du and Groover, 2010; Yamaguchi et al., 2010; Zhong et al., 2010; Nakano et al., 2015; Kumar et al., 2016). This delicate network ensures a differential regulation of the formation of diverse SCW with varied components and thicknesses in secondary xylem under various conditions (Zhou et al., 2009; Zhong et al., 2010; Kumar et al., 2016). For example, *PsnSHN2* (Liu et al., 2017) and *AtSHN2* (Ambavaram et al., 2011), two higher hierarchical master switches of this network, significantly increase cellulose and hemicellulose contents and SCW thickness with significantly decreased lignin content through activating or repressing their downstream TFs and structural genes in overexpression of tobacco and rice, respectively. In addition, other higher hierarchical regulators that have been reported include *PtrGATA12* and *PtrHAT22* (Ren et al., 2021, 2022). For this reason, we need to continuously identify more higher hierarchical regulators that may be useful for leveraging the manipulation of important traits.

In this report, we revealed that overexpression of *PtrWOX13A* (Potri.005G101800.1), a member of the ancient clade of *Populus trichocarpa WOX* family, manifested not only the significantly enhanced growth potentials but also notably altered SCW and fiber characteristics, accompanied by the expression-level changes of the *TFs* in different hierarchical layers and the structural genes. The bioactive GA contents were elevated presumably due to the upregulation of three key genes involved in GA activation in transgenic poplar. PtrWOX13A was shown to be capable of directly activating some SCW-associated *TFs*, structural genes, *PtrGA20ox4*, and *PtrGA3ox1* through binding to two *cis*-elements that are presented in the promoters of these genes. Collectively, our findings suggest that *PtrWOX13A* is a higher hierarchic regulator that regulates some wood-formation TFs, structural genes, and/or through triggering GA-mediated signaling cascade. The *PtrWOX13A* and its target genes identified in this study will be instrumental in developing new strategies for genetic improvement of wood quality.

## Materials and Methods

### Plant Materials

The plantlets of *P. trichocarpa* clone Nisqually-1 were obtained from the Center for Excellence in Molecular Plant Sciences, Chinese Academy of Sciences, and vegetatively propagated in our laboratory through tissue culture (Ren et al., 2021).

In the Northeast Forestry University greenhouse, 1-year-old *P. trichocarpa* trees were propagated and planted in a mixture of turfy peat and sand (2:1 v/v) and grown for 90 days under 16 h day/8 h night photoperiod at 25°C. The roots, primary leaves, transition leaves, secondary leaves, primary phloem, transition phloem, secondary phloem, primary xylem, transition xylem, and secondary xylem were collected from the above plants and immediately frozen in liquid nitrogen and stored at −80°C. Tissue culture seedlings that were 1-month old were treated with GA_3_ (1 μM), and the samples were collected after treatment for 0, 3, 6, 12, 24, 48, 72, and 96 h, respectively. The RNA was isolated according to the method described in a previous report (Liao et al., 2004) and subsequently treated with DNase I (Qiagen) to remove genomic DNA.

### Sequence Comparisons, Protein Sequence, and Promoter Analysis

Multiple sequence alignments were carried out using ClustalW2^1^ with default setting (Larkin et al., 2007). The homologous gene sequences from *Populus trichocarpa* [Potri.005G101800.1 (*PtrWOX13A*), Potri.002G008800.1 (*PtrWOX13B1*), and Potri.005G252800.1 (*PtrWOX13B*)] and *Arabidopsis thaliana* [AT4G35550.1 (*AtWOX13*)] were retrieved from Phytozome Database^2^ using TBLASTP with the *AtWOX13* protein sequence as the query sequence.

The conserved motifs in the PtrWOX13 protein were analyzed by Multiple Expectation Maximization for Motif Elicitation (MEME) v. 5.3^3^ with default parameters (Bailey et al., 2006). Conserved motifs were identified with the motif widths of 6-50 residues.

Sequences of 2,000 nucleotides before the transcription start codon were extracted from the genomic sequences of the *PtrWOX13A*, *PtrWOX13B1*, and *PtrWOX13B* from Phytozome (see the footnote 2), and the *cis*-regulatory elements were predicted by the program PlantCARE online^4^ (Lescot et al., 2002).

### Cloning *PtrWOX13A* From *P. trichocarpa*

The full *PtrWOX13A* (Potri.005G101800.1) cDNA was amplified with gene-specific primers (Supplementary Table 1) using the method described in a previous report (Liu et al., 2017), and then transformed into *Escherichia coli* cells (DH5α, TsingKe) for validation by Sanger sequencing.

### Subcellular Localization

The full-length coding region of *PtrWOX13A* without termination codon was amplified using specific primers (Supplementary Table 1) and then fused to the N-terminal of GFP under the control of *CaMV 35S* promoter in the pBI121 vector. The two fusion constructs were delivered into onion epidermal cells *via* particle bombardment. The GFP fluorescent images were photographed with confocal microscopy (Leica TCS SP5) 48 h after the bombardment with a gene gun (GJ-1000).

### Transcriptional Activation Assay

The transcriptional activation of PtrWOX13A on putative target genes was tested using the yeast two–hybrid system. The complete CDS of *PtrWOX13A* was amplified using specifically designed primers (Supplementary Table 1). The amplified fragments were fused in-frame to the pGBKT7 vector to generate the pGBKT7*-PtrWOX13A* construct. The pGBKT7*-PtrWOX13A* and the pGBKT7 blank vector (as negative control) were transformed into Y2H yeast cells independently. The transformed Y2H yeast cells were plated onto SD/-Trp (growth control), SD/-Trp/-His/-Ade, and X-α-Gal media and incubated at 30°C for 3–5 days to identify the PtrWOX13A transcriptional activation.

### Transformation of *P. trichocarpa* for Generating *PtrWOX13A* Overexpression Lines

The *PtrWOX13A* was amplified with specific primers (Supplementary Table 1) and then inserted into the pROKII vector. The pROKII*-PtrWOX13A* was first transferred into *Agrobacterium tumefaciens* GV3101 using the freeze–thaw method. The transgenic method was described as the previous report (Alvarez et al., 2018). The genomic DNA of all kanamycin-resistant shoots amplified by regular PCR using the PROKII sequencing primers is listed in Supplementary Table 1 to verify whether *PtrWOX13A* was integrated into the poplar genome.

The verified *PtrWOX13A* transgenic lines and wild-type (WT) poplars were propagated and planted in a mixture of turfy peat and sand (2:1 v/v), and grown under 16 h/8 h day/night photoperiod at 25°C in the greenhouse. After the *PtrWOX13A* transgenic lines were grown for 90 days, they were used for further characterization assays.

### Phenotypic Trait Measurement

The phenotypic traits, which included lengths and widths of leaves, heights, base diameters, fresh and dry weights, and breaking forces of stems, of wild type (WT) and *PtrWOX13A* transgenic lines were measured following the method described in our previous publication (Ren et al., 2021).

### Fiber Measurements

Pieces of outer xylem (approximately 1 mm × 1 cm × 0.5 mm) from Internode 8 (IN8) of the plants used for the anatomical characterization were macerated to separate the wood cells. The cells were observed under an Axioplan 2 microscope (Zeiss), and lengths and diameters of 100 fibers per plant were measured using AxioVision 4.6 software.

### Determination of Gibberellin Contents

Fresh stems between IN5 and IN8 of *PtrWOX13A* transgenic lines and WT were used to measure GA_1_ and GA_4_ contents. The contents of GAs were measured with high-performance liquid chromatography (HPLC) by Suzhou Michy Biomedical Technology Co., Ltd., in China (Yang et al., 2014). Values are means ± SD of three biological replicates of three individual plants.

### Scanning Electron Microscopy and Histological Analysis

Stem segments were prepared by freeze-drying for measuring SCW thickness using scanning electron microscopy (SEM) (S-4800, HITACHI) following the method described in a previous report (Liu et al., 2017). The SCW thicknesses of fibers in the SEM micrographs were quantified in a randomly selected area of 45 cells using ImageJ software^5^.

### Histological Analysis and Determination of Cellulose, Hemicellulose, and Lignin Contents

The histological analysis was carried out with a Laser Scanning Confocal Microscope (LSM800) and the determinations of lignin, cellulose, and hemicellulose contents were conducted using the ANKOM 2000i Automatic Fiber Analyzer (ANKOM). Both of these analyses can be found in our previous studies (Liu et al., 2017).

### Gene Expression Analysis of Poplar

RNA from the xylem of stems, spanning IN5 to IN8 of *PtrWOX13A* transgenic lines and WT, were used for synthesizing cDNA. Samples of cDNA were run in triplicate using the Applied Biosystems 7500 Real-Time PCR System to determine the critical threshold (Ct) with the SYBR Premix Ex Taq kit (TaKaRa). Then, the relative gene expression levels were calculated.

The primers used for the analysis of the expression levels of the *PtrWOX13A*, *PtrWOX13B1*, and *PtrWOX13B2* by qRT-PCR are listed in Supplementary Table 2. The expression levels of genes involved in cellulose biosynthesis (*PtrCESA4*, *PtrCESA7*, and *PtrCESA8*) (Suzuki et al., 2006), xylan biosynthesis (*PtrGT43A*, *PtrGT47C*, and *PtrGT8F*) (Zhou et al., 2006, 2007), lignin biosynthesis (*PtrPAL4*, *PtrC4H1*, *PtrC3H3*, *Ptr4CL5*, *PtrCCoAOMT3*, *PtrCOMT2*, *PtrCCR2*, *PtrCAld5H2*, and *PtrCAD1*), lignin polymerization (*PtrLAC19*, *PtrLAC21*, *PtrLAC26*, and *PtrLAC41*) (Marjamaa et al., 2009; Lu et al., 2013), fiber elongation (*PtrCSLD2*, *PtrXTH5*, *PtrEXPA8*, and *PtrFRA2*) (Burk et al., 2001; Gray-Mitsumune et al., 2004; Samuga and Joshi, 2004; Esmon et al., 2006; Seale, 2020), the TFs belonging to well-known hierarchical transcription regulatory network governing poplar SCW formation (*PtrWND6A*, *PtrWND6B*, *PtrMYB20*, *PtrMYB21*, *PtrMYB157*, *PtrMYB28*, *PtrMYB152*, *PtrMYB52*, and *PtrMYB54*) (Zhong et al., 2010, 2011; Zhong and Ye, 2010; Hussey et al., 2013; Zhang et al., 2018; Cubria-Radio and Nowack, 2019; Zhang J. et al., 2020), and genes involved in homeostasis of bioactive GAs (GA_4_ and GA_1_) (*PtrGA20ox4*, *PtrGA3ox1*, *and PtrGA2ox1*) (Israelsson et al., 2005) were analyzed by qRT-PCR using specific primers (Supplementary Table 2). *PtrActin* was employed as internal control, and the delta-delta CT method was used to quantify gene expression levels relative to *PtrActin* as described in a previous report (Zhang Y. et al., 2020).

### Transient Expression Assay

The full coding region of *PtrWOX13A* was cloned into pROKII under the control of the *CaMV 35S* promoter, which was used as the effector construct. The reporter construct contained the *GUS* reporter gene driven by the various 2-kb promoters of genes, which were chosen based on the changes in the expression levels in *PtrWOX13A* transgenic lines as compared with WT. The effector vector, reporter vector, and 35S-LUC-pGreenII 8000 vector transfected tobacco leaves together. GUS activity in tobacco leaves transfected with pROKII empty vector (without *PtrWOX13A*), and reporter vector (with one of the selected promoters), and 35S-LUC-pGreenII 8000 were used as a control. The 35S-LUC was an internal baseline control for the transient expression analysis to use for the normalization of GUS activity. Each promoter was amplified using the primers listed in Supplementary Table 3 designed from *P. trichocarpa* genomic DNA. The GUS activity was measured by the method described in our previous study (Ren et al., 2021). GUS activity for each promoter tested is expressed as the ratio of GUS/LUC obtained with the effector pROKII-PtrWOX13A to GUS/LUC obtained with the control effector pROKII empty vector.

### Analysis of the Downstream *Cis*-Regulatory Elements of the *PtrWOX13A*

Three tandem copies of two *cis*-regulatory elements, ATTGATTG and TTAATSS (Supplementary Table 4), were fused to the multiple cloning site upstream of the HIS3 reporter gene in pHIS2. The full CDS of *PtrWOX13A* amplified with the primers in Supplementary Table 1 was inserted into pGADT7-Rec2 as the effector vector. Both constructs were co-transformed into Y187 yeast cells, which were plated onto DDO, TDO, and TDO plus 60 mM 3-aminotriazole to test the expression of the *His3* gene at 30°C for 3–5 days. The pair of pHIS2*-p53* and pGADT7-*rec2*-*p53* was used as a positive co-transformation control, whereas the pair of pHIS2*-p53* and pGADT7-*rec2-PtrWOX13A* was used as a negative control. The interactions of these sequences with *PtrWOX13A* were studied using the yeast one-hybrid analysis (Y187). The ExactSearch tool was employed to identify these motifs in the promoter regions of putative target genes we identified (Supplementary Table 5; Gunasekara et al., 2016).

### ChIP-PCR

For immunoprecipitation analysis, 1-month-old *35-PtrWOX13A-Flag* overexpressing transgenic seedlings that had been subjected to tissue culture were harvested and vacuumed for 15 min in 10 ml buffer solution, followed by the addition of 4 ml 2M glycine to quench cross-linking by and then vacuumed for 2 min. The treated plants were washed with H_2_O three times and ground in liquid nitrogen. Immunoprecipitation was performed with anti-FLAG antibody according to Zhao et al. (2020). PCR was used to determine the binding sites of *PtrWOX13A* in the promoter regions. Primers are listed in Supplementary Table 6. All experiments were performed in three biological replications with three technical replicates.

### Statistical Analysis

The Dunnett’s test (SPSS 17.0) was used to test the statistical significance of the data. The difference between two groups of data for comparisons in this study was evaluated by significance (*p < 0.05) or high significance (**P < 0.01).

## Results

### Identification and Characterization of *PtrWOX13*

The previous phylogenetic analysis revealed that the ancient clade of *Arabidopsis* and poplar WOX families include three *Arabidopsis* and three poplar WOX genes (Liu et al., 2014). The ortholog counterparts of *AtWOX10* and *AtWOX14* in poplar were lost, while the ortholog counterpart of *AtWOX13* in poplar was evolved into three paralogous genes that include *PtrWOX13A* (Potri.005G101800.1), *PtrWOX13B1* (Potri.002G008800.1), and *PtrWOX13B2* (Potri.005G252800.1) (Supplementary Figure 1A).

Multiple sequence alignment of AtWOX13 and three PtrWOX13 protein sequences showed that the three PtrWOX13 proteins contained not only the typic HD domain consisting of three α-helices separated by a loop and a turn but also the following conservative features unique to WOX ancient clade: (1) the N-terminal domain located in the upstream of the HD domain (Sakakibara et al., 2014); (2) the ESExE motif located in the downstream of the HD domain (Deveaux et al., 2008); (3) the WOX13 MOG motif consisting of 31 amino acids that are found upstream of the HD domain (Wu et al., 2019); (4) the YxDpl motif between the WOX13 MOG motif and the HD domain (Deveaux et al., 2008); and (5) the signature motif of the ancient clade consisting of NVYNWFQNR (Wu et al., 2019; Supplementary Figure 1B).

In addition, the results of the conserved motif analysis of the three PtrWOX13 proteins suggested that PtrWOX13B1 and PtrWOX13B2 proteins shared all five aforementioned domain/motifs while PtrWOX13A had no motif 5 (Supplementary Figure 2A). Moreover, the similarity of protein sequences was only 54.8% between the PtrWOX13A and PtrWOX13B1, 53.4% between PtrWOX13A and PtrWOX13B2, but 86.1% between PtrWOX13B1 and PtrWOX13B2. In summary, these data suggested that PtrWOX13A has evolved to be more distal to both PtrWOX13B1 and PtrWOX13B2, and thus may be functionally distinct from both of them.

To further determine if there were differential responses to environmental and intracellular cues among the three *PtrWOX13* genes, we analyzed the *cis*-regulatory elements in their promoters. The results showed that there were 11 common *cis*-regulatory elements, for example, MYC, MYB, MBS, ARE, and ABRE among all three genes (Supplementary Figures 2B,C). In addition, there were 20 common *cis*-regulatory elements between *PtrWOX13B1* and *PtrWOX13B2* promoters, 13 between *PtrWOX13A* and *PtrWOX13B1* promoters, and 14 between *PtrWOX13A* and *PtrWOX13B1* promoters (Supplementary Figure 2C), which indicates that responses of *PtrWOX13B1* and *PtrWOX13B2* appear to be more similar among the three *PtrWOX13* genes. Moreover, each promoter of these genes also had unique *cis*-regulatory elements. For example, there were P-box, TGA-element, and TCA-element that were only present in the *PtrWOX13A* promoter; G-Box, ACE, and AAGAA-motifs that were only present in the *PtrWOX13B1* promote; and RY-element, TC-rich repeats, and LTR that were only present in the *PtrWOX13B2* promoter (Supplementary Figure 2C). These data further suggested that these three *PtrWOX13* genes in poplar have subfunctionalized after duplication.

**FIGURE 1:**
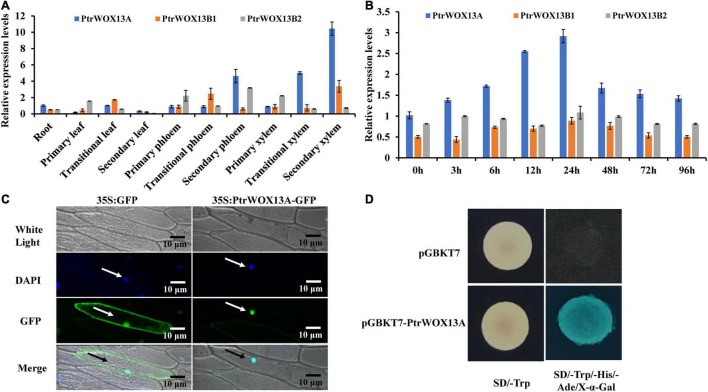
Tissue-specific expression patterns, subcellular localization, and transcriptional activity of *PtrWOX13A*. **(A)** The tissue-specific expression patterns of three *PtrWOX13* genes as determined by qRT-PCR analysis. *PtrActin* was used as a reference gene. The expression of *PtrWOX13A* in root was set to 1 and expression in all other tissues is relative to roots. **(B)** The temporal expression patterns of three *PtrWOX13* genes after GA treatments as determined by qRT-PCR analysis. *PtrActin* was used as a reference gene. The expression of *PtrWOX13A* at 0 h was set to 1, all other time points were relative to 0 h. **(C)** Subcellular localization of PtrWOX13A. Confocal images manifested the localization of PtrWOX13A: DAPI, a nuclear staining dye; GFP proteins in the nuclei of onion epidermal cells. Merge: The merged images of White light, DAPI, and GFP staining. Arrows indicate cells located in the epidermis of onions. **(D)** Transcriptional activation analysis of PtrWOX13A fused with the GAL4 DNA binding domain (GAL4DB) in yeast showed its potential to activate the expression of the His-3 and X-α-Gal reporter genes. Arrows indicate cells located in the epidermis of onions.

To further determine which of the three *PtrWOX13* genes participated in wood formation, we analyzed their expression levels in various tissues of poplar by quantitative RT-PCR (qRT-PCR). As shown in Figure 1A, the three *PtrWOX13* genes were differently expressed at detectable levels in all examined tissues. Among them, the transcript levels of *PtrWOX13A* in the transition and secondary xylem were much higher than those in any other tissues and those of the other two *PtrWOX13* transcripts in the same tissues, indicating that *PtrWOX13A* was the most likely gene to participate in wood formation among the three *PtrWOX13* genes (Figure 1A). In addition, given that GAs have important roles in wood formation and *PtrWOX13A* has GAs response *cis*-element, a P-box, in its promoter, we further tested if the *PtrWOX13* was inducible in the secondary tissues of poplar stems by exogenous GA treatment. The transcript levels of *PtrWOX13A* were evaluated by qRT-PCR after treatment with exogenous GAs using GA_3_. The results show that the expression of *PtrWOX13A* increased after treatment with exogenous GA_3_ for 24 h, and then decreased from 24 to 96 h, whereas the transcriptional levels of *PtrWOX13B1* and *PtrWOX13B2* had no obvious changes in response to exogenous GA treatment (Figure 1B). Thus, we chose *PtrWOX13A* for studying the functions of WOX genes in wood formation.

### Subcellular Localization and Transcriptional Activation Activity of PtrWOX13A

To determine whether PtrWOX13A localized in the nuclei, we performed the transient expression of *PtrWOX13A* in onion epidermal cells using the particle gun bombardment method. As visualized with a fluorescence confocal microscope, the PtrWOX13A–GFP fusion protein was exclusively colocalized to DAPI-stained nuclei (Figure 1C), indicating that PtrWOX13A was a nuclear-localized TF.

In addition, the presence of an acidic amino-terminal domain next to an HD domain in the C-terminal region, as seen in Supplementary Figure 1B, suggested that PtrWOX13A was a transcriptional activator. To verify this, we fused PtrWOX13A with the GAL4 DNA-binding domain and tested its potential to activate the reporter gene expression in yeast. As shown in Figure 1D, PtrWOX13A activated the expression of His3 and X-α-Gal reporter genes, indicating that it was indeed a transcriptional activator.

### Phenotypic Changes of the *PtrWOX13A* Overexpression Transgenic Lines

To investigate the functions of *PtrWOX13A* in poplar, we expressed *PtrWOX13A* under the control of the 35S promoter in WT. In total, eight transgenic lines were generated and corroborated to harbor the transformed *PtrWOX13A* by genomic PCR (Supplementary Figure 3A). Then, the expression levels of *PtrWOX13A* in these transgenic lines were quantitatively determined using qRT-PCR. Three transgenic lines, OE-2, OE-4, and OE-7, which had higher expression levels of *PtrWOX13A* compared with other transgenic lines, were chosen for further characterization of *PtrWOX13A*’s functions (Supplementary Figure 3B).

**FIGURE 2:**
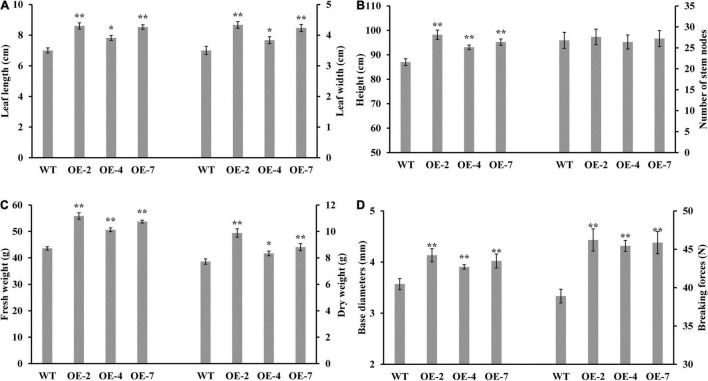
Effect of *PtrWOX13A* overexpression on growth-related traits in *Populus trichocarpa* transgenic lines. **(A)** Average leaf length and leaf width; **(B)** Average height and number of stem nodes and standard error of each transgenic line; **(C)** Average fresh weight and dry weight; **(D)** Average base diameters and breaking forces. All the data were measured on the 90-day-old wild type (WT) and *PtrWOX13A* transgenic lines (OE-2, OE-4, and OE-7). Each error bar represents the standard deviation of three biological replicates. Asterisks denote levels of significance (Dunnett’s test; *p < 0.05 significant, and **p < 0.01, highly significant).

The three *PtrWOX13A* transgenic lines all exhibited significantly enhanced growth potentials compared with WT. For example, the lengths and widths of leaf, heights, base diameters, and fresh and dry weights of the three *PtrWOX13A* transgenic lines increased by 18.8, 18.1, 9.7, 12.6, 22.4, and 16.6% on average as compared with those of the WT, respectively (Figures 2A–D). In addition, the breaking forces of stems, which is the important physical property of wood, increased 17.8% on average in *PtrWOX13A* transgenic lines compared to WT (Figure 2D). These data indicate that *PtrWOX13A* overexpression results in enhanced vegetative growth and increased biomass productivity of transgenic lines.

### Effects of *PtrWOX13A* Overexpression on Wood Characteristics and Bioactive Gibberellin Homeostasis in Transgenic Poplar

To investigate the effects of *PtrWOX13A* overexpression on poplar wood formation, we performed histochemical staining of the stem cross-sections of *PtrWOX13A* transgenic lines. The toluidine blue and phloroglucinol-HCl were used to stain cell morphology and lignin, respectively. As shown in Supplementary Figure 4, the staining intensities of the cross-sections from IN2 showed no obvious difference between the *PtrWOX13A* transgenic lines and WT, but the staining intensities of the cross-sections in the IN4, IN6, to IN8 exhibited increasingly strong staining signals in *PtrWOX13A* transgenic lines compared to those of WT, which implicates that *PtrWOX13A* enhances wood lignification during stem development in the transgenic lines.

**FIGURE 3:**
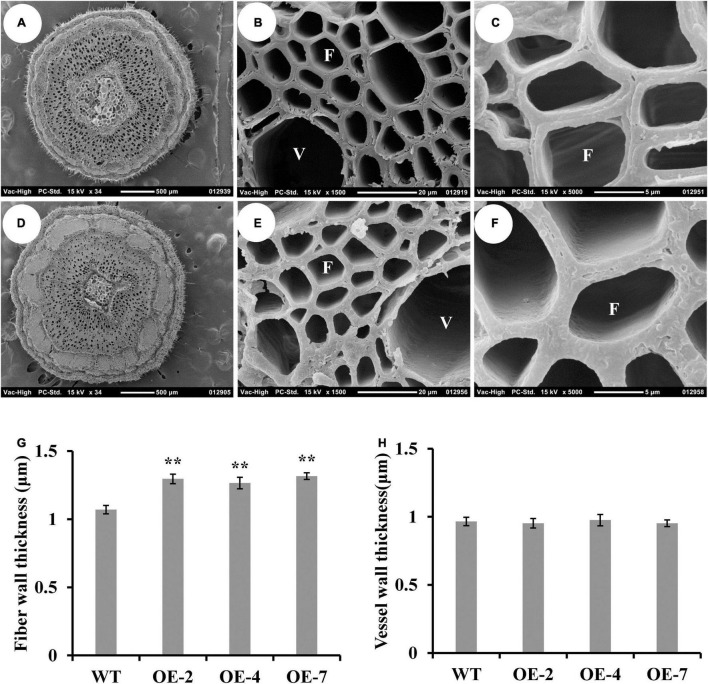
Effect of *PtrWOX13A* overexpression on the secondary wall thickness of stems in *Populus trichocarpa*. **(A–F)** The scanning electron microscope of cross stem sections of wild type **(A–C)** and PtrWOX13A overexpression transgenic lines **(D–F)**. **(A,D)** represent 34×, **(B,E)** represent 1,500×, and **(C,F)** represent 5,000× scanning electron microscope of cross stem sections, respectively. V and F in **(B,C,E,F)** represent vessel cell and fiber cell, respectively. **(G)** The fiber wall thicknesses of cross stem sections in wild type (WT) and *PtrWOX13A* overexpression transgenic lines (OE-2, OE-4, and OE-7). **(H)** The vessel wall thicknesses of cross stem sections in WT and *PtrWOX13A* overexpression transgenic lines. Error bars represent the standard deviation of three biological replicates. Asterisks indicate levels of significance (Dunnett’s test; **p < 0.01). All data were measured on the 8th stem of 90-day-old poplars.

Then, we further analyzed the wood characteristics in the IN8 of *PtrWOX13A* transgenic lines. The scanning electron microscope of stem cross-sections revealed that the average SCW thicknesses of fibers increased by 20.8% on average, while the average SCW thickness of vessels had little or no changes in the *PtrWOX13A* transgenic lines compared to that of the WT (Figures 3A–H). These results indicate that overexpression of *PtrWOX13A* has different effects on the fibers and vessel formation in the transgenic poplars.

**FIGURE 4:**
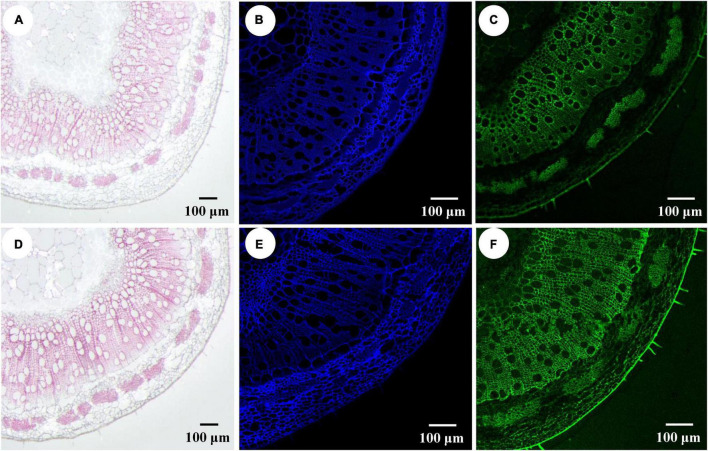
Impact of *PtrWOX13A* overexpression on components of the secondary cell wall of stems in *Populus trichocarpa.* **(A)** Stem section of wild type stained with Phloroglucinol-HCl (red color). **(B)** Stem section of wild type stained with Calcofluor white staining (blue color). **(C)** Stem section of wild type stained with Monoclonal Antibody LM10 (green color). **(D)** Stem section of *PtrWOX13A* overexpression transgenic lines stained with Phloroglucinol-HCl (red color). **(E)** Stem section of *PtrWOX13A* overexpression transgenic lines stained with Calcofluor white (blue color). **(F)** Stem section of *PtrWOX13A* overexpression transgenic lines stained with Monoclonal Antibody LM10 (green color). Scale bars = 100 μm. All data were measured on the 8th stem of 90-day-old poplars.

**FIGURE 5:**
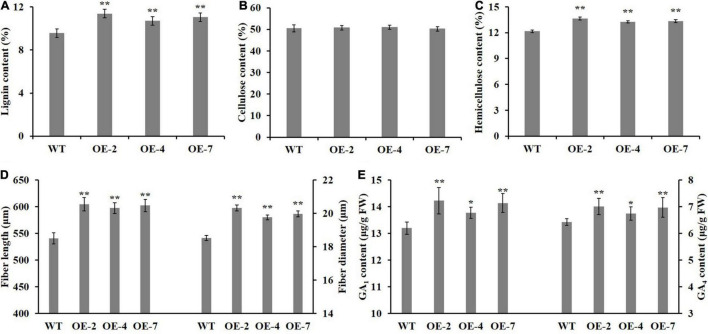
Effect of *PtrWOX13A* overexpression on secondary cell wall of stems in *Populus trichocarpa*. **(A–E)** Represent lignin content, cellulose content, hemicellulose content, fiber length, fiber diameter, GA_1_ content, and GA_4_ content, respectively. Error bars represent the standard deviation of three biological replicates. Asterisks indicate levels of significance (Dunnett’s test; *p < 0.05 significant, and **p < 0.01, highly significant).

To determine which component (e.g., cellulose, hemicellulose, or lignin) contributed more to fiber SCW thickening, the phloroglucinol-HCl and calcofluor white were used to stain lignin and cellulose, respectively, and the monoclonal antibody LM10 was used to label hemicellulose (xylan) immunologically. As shown in Figure 4, the widths of staining zones of phloroglucinol-HCl (Figure 4A) and antibody LM10 calcofluor (Figure 4C) in *PtrWOX13A* transgenic lines were larger than those in WT, while the width of staining zone of calcofluor white in *PtrWOX13A* transgenic lines (Figure 4B) was not larger than that in WT (Figures 4A–F). In addition, the staining intensities of both dyes and antibodies had stronger intensities in transgenic lines than in WT. To further accurately assess these changes in SCW components, we analyzed the three main components of SCW using the automatic fiber analyzer. The results revealed that the lignin and hemicellulose contents significantly increased by 15.3 and 10.2%, respectively, on average, while the cellulose contents in *PtrWOX13A* transgenic lines had no significant changes compared with that in WT (Figures 5A–C). These results indicate that *PtrWOX13A* overexpression results in increased contents of lignin and hemicellulose but had no obvious effects on the cellulose deposition in transgenic lines compared with WT. In addition, *PtrWOX13A* transgenic lines exhibited notably increased fiber length and base diameter by 11.3 and 8.1% on average, respectively, as compared with WT (Figure 5D).

Given that *AtWOX14* is involved in bioactive GA homeostasis (Denis et al., 2017), we suspected that *PtrWOX13A*, a member of the WOX ancient clade that includes *AtWOX14*, also affected the bioactive GA homeostasis in its overexpression transgenic poplars. The analysis of the bioactive GAs showed that the contents of bioactive GA_1_ and GA_4_ increased by 6.39 and 7.52%, respectively, on average in the IN5-8 of *PtrWOX13A* transgenic lines compared with WT (Figure 5E). Taken together, these results implicate that *PtrWOX13A* participates in wood formation and bioactive GA homeostasis of transgenic poplar.

### Overexpression of *PtrWOX13A* Altered the Expressions of Genes Involved in Secondary Cell Wall and Fiber Formation and Bioactive Gibberellin Homeostasis in Poplar

**FIGURE 6:**
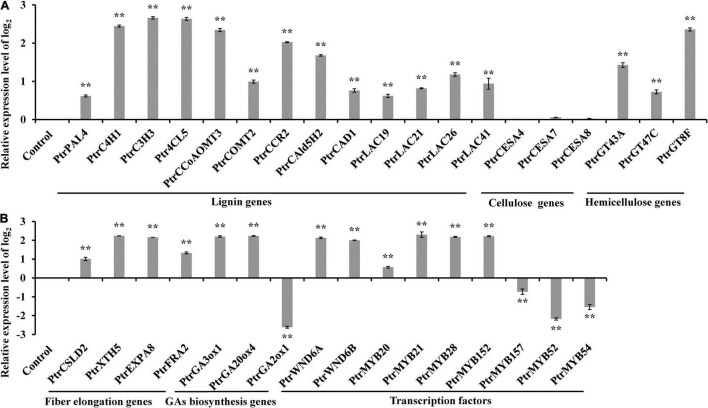
Expression analysis of wood formation pathway genes and their regulatory genes in 90 days old wild-type and *PtrWOX13A* transgenic lines. **(A)** Represents expression analysis of lignin biosynthesis genes, cellulose biosynthesis genes, and hemicellulose biosynthesis genes. **(B)** Represents expression of fiber cell elongation genes, GA biosynthesis genes, and TFs. PtrActin was used as a reference gene. Control represents the normalized expression level (namely 0 in this case) of each gene examined in wild type plants. Each error bar represents the standard deviation of three biological replicates. Asterisks indicate levels of significance (Dunnett’s test; **p < 0.01, highly significant). Those genes include lignin biosynthesis genes (*PtrPAL4*, *PtrC4H1*, *PtrC3H3*, *Ptr4CL5*, *PtrCCoAOMT3*, *PtrCOMT2*, *PtrCCR2*, *PtrCAld5H2*, and *PtrCAD1*), lignin polymerization genes (*PtrLAC19*, *PtrLAC21*, *PtrLAC26*, and *PtrLAC41*), cellulose biosynthesis genes (*PtrCESA4*, *PtrCESA7*, and *PtrCESA8*), hemicellulose biosynthesis genes (*PtrGT43A*, *PtrGT47C*, and *PtrGT8F*), fiber cell elongation genes (*PtrCSLD2*, *PtrXTH5*, *PtrEXPA8*, and *PtrFRA2*), GA biosynthesis genes (*PtrGA20ox4*, *PtrGA3ox1*, and *PtrGA2ox1*), and TFs (*PtrWND6A*, *PtrWND6B*, *PtrMYB20*, *PtrMYB21*, *PtrMYB28*, *PtrMYB152*, *PtrMYB157*, *PtrMYB52*, and *PtrMYB54*).

To uncover the molecular mechanism underlying the aforementioned changes in secondary growth and bioactive GA homeostasis, we analyzed the expression levels of the genes related to these changes in the *PtrWOX13A* transgenic lines. The qRT-PCR results demonstrated that the expression levels of lignin biosynthesis genes including *PtrPAL4*, *PtrC4H1*, *PtrC3H3*, *Ptr4CL5*, *PtrCCoAOMT3*, *PtrCOMT2*, *PtrCCR2*, *PtrCAld5H2*, and *PtrCAD1*, lignin polymerization genes including *PtrLAC19*, *PtrLAC21*, *PtrLAC26*, and *PtrLAC41*, and hemicellulose biosynthesis genes including *PtrGT43A*, *PtrGT47C*, and *PtrGT8F*, increased at extremely significant levels, while the expression levels of the cellulose biosynthesis genes such as *PtrCESA4*, *PtrCESA7*, and *PtrCESA8*, had no obvious increases in *PtrWOX13A* transgenic lines compared with WT (Figure 6A). In addition, the genes related to fiber cell elongation, such as *PtrCSLD2*, *PtrXTH5*, *PtrEXPA8*, and *PtrFRA2*, were notably upregulated in *PtrWOX13A* transgenic lines compared with WT. Moreover, we observed that the bioactive GA biosynthesis genes such as *PtrGA20ox4* and *PtrGA3ox1* were significantly upregulated, whereas the expression levels of *PtrGA2ox1*, a bioactive GA deactivation gene, significantly decreased in *PtrWOX13A* transgenic lines compared with WT (Figure 6B).

It was noteworthy that the expression levels of the master switches of SCW formation, such as *PtrWND6A*, *PtrWND6B*, *PtrMYB20*, and *PtrMYB21*, significantly increased in *PtrWOX13A* transgenic lines compared with WT (Figure 6B). In addition, the expression levels of *TFs* that are capable of activating lignin biosynthesis, such as *PtrMYB28* and *PtrMYB152*, also increased significantly. On the contrary, the expression levels of *TFs* that are capable of repressing lignin biosynthesis, such as *PtrMYB157*, *PtrMYB52*, and *PtrMYB54*, significantly decreased in *PtrWOX13A* transgenic lines compared with WT (Figure 6B). These data aligned well with the changes in SCW and fiber characteristics observed in *PtrWOX13A* transgenic lines.

### PtrWOX13A Activated the Promoters of the Genes Involved in Wood Formation and Bioactive Gibberellin Homeostasis

**FIGURE 7:**
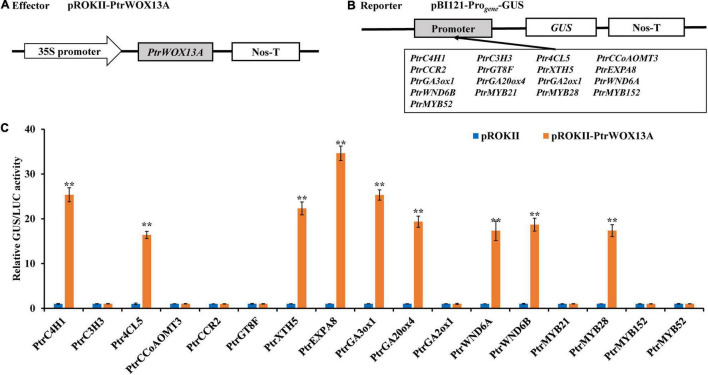
Activation of the promoters of poplar transcription factors and secondary cell wall biosynthesis pathway genes by *PtrWOX13A*. **(A,B)** Diagrams of the effector and the reporter constructs. **(C)** The expression of the GUS under the control of those promoters that were activated by *PtrWOX13A*. GUS activity in tobacco leaves transfected with pROKII empty vector, reporter vector, and 35S-LUC-pGreenII 8000 vector was used as a control and was set to 1. GUS activity for each promoter tested is expressed as the ratio of GUS/LUC obtained with the effector pROKII-PtrWOX13A to GUS/LUC obtained with the control effector pROKII empty vector. Error bars represent the standard deviation of three biological replicates. Asterisks indicate levels of significance of differential expression (Dunnett’s test; **p < 0.01, highly significant). Those genes include lignin biosynthesis genes (*PtrC4H1*, *PtrC3H3*, *Ptr4CL5*, *PtrCCoAOMT3*, and *PtrCCR2*), hemicellulose biosynthesis genes (*PtrGT8F*), fiber cell elongation genes (*PtrXTH5* and *PtrEXPA8*), GA biosynthesis genes (*PtrGA20ox4*, *PtrGA3ox1*, and *PtrGA2ox1*), and TFs (*PtrWND6A*, *PtrWND6B*, *PtrMYB21*, *PtrMYB28*, *PtrMYB152*, and *PtrMYB52*).

Based on the fact that overexpression of *PtrWOX13A* significantly altered the expression of several genes in transgenic poplar, we investigated whether it could activate these genes directly rather than indirectly, whose expression levels had a two-fold change in *PtrWOX13A* transgenic lines compared with WT, by conducting transient expression assays in tobacco leaves. The 2-kb proximal promoter regions of these genes, upon being amplified from *P. trichocarpa* genomic DNA, were linked to the GUS reporter gene to create the reporter constructs, and the full-length cDNA of *PtrWOX13A* was ligated to the immediately downstream of 35S promoter to generate the effector construct (Figures 7A,B). The reporter and effector constructs were co-transfected into tobacco leaves by the *Agrobacterium*-mediated method. The subsequent assay of the GUS activity demonstrated that PtrWOX13A significantly activated the lignin biosynthesis genes of *PtrC4H1* and *Ptr4CL5*, the fiber cell elongation genes of *PtrXTH5* and *PtrEXPA8*, GA biosynthesis genes of *PtrGA20ox4* and *PtrGA3ox1*), and TFs of *PtrWND6A*, *PtrWND6B*, and *PtrMYB28* albeit to different levels (Figure 7C), Contrastingly, PtrWOX13A did not activate or inhibit lignin biosynthesis genes of *PtrC3H3*, *PtrCCoAOMT3*, and *PtrCCR2*), hemicellulose biosynthesis genes of *PtrGT8F*, GA biosynthesis genes of *PtrGA2ox1*, and TFs of *PtrMYB21*, *PtrMYB152*, and *PtrMYB52* (Figure 7C). These data suggest that PtrWOX13A directly regulates some genes involved in wood formation and bioactive GA homeostasis through binding to their promoters, while some other significantly changed genes were supposedly regulated indirectly.

### PtrWOX13A Bound to Two *Cis*-Regulatory Elements in the Promoters of Its Directly Regulated Genes

The key mechanism for a TF to directly regulate downstream genes depends on the sequence-specific binding site to DNA motifs present in the promoters of its target genes. Thus, we further performed the yeast one-hybrid assay to test if PtrWOX13A could bind to the aforementioned *cis*-regulatory elements, ATTGATTG and TTAATSS (Minh-Thu et al., 2018; Ren et al., 2022). In addition, we found that promoter regions of the genes, such as *PtrC4H1*, *Ptr4CL5*, *PtrXTH5*, *PtrEXPA8*, *PtrGA20ox4*, *PtrGA3ox1*, *PtrWND6A*, *PtrWND6B*, and *PtrMYB28*, also contain these two *cis*-regulatory elements (Supplementary Table 5). As shown in Supplementary Figure 5, PtrWOX13A had obvious binding affinities to ATTGATTG (denoted by A in the figure) and TTAATSS (denoted by T in the figure) *cis*-regulatory elements.

**FIGURE 8:**
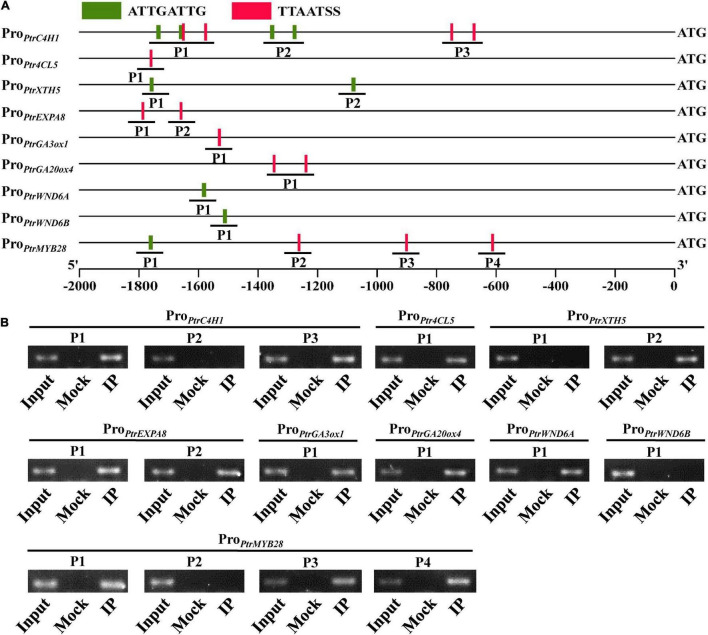
*PtrWOX13A* directly binds to the promoters of the poplar transcription factors and secondary cell wall biosynthesis pathway genes. **(A)** Schematic diagrams of the promoters showing the locations amplified by ChIP-PCR. **(B)** ChIP-PCR analysis of the promoter fragments enriched due to *PtrWOX13A* binding during immunoprecipitation; 30-day-old poplar plants overexpressing PtrWOX13A-Flag used for ChIP with anti-Flag antibodies. Input, Mock, and IP indicate the chromatin before immunoprecipitation (IP), IP with no antibodies, and IP with anti-FLAG antibodies, respectively. Those genes include lignin biosynthesis genes (*PtrC4H1* and *Ptr4CL5*), fiber cell elongation genes (*PtrXTH5* and *PtrEXPA8*), GA biosynthesis genes (*PtrGA20ox4* and *PtrGA3ox1*), and TFs (*PtrWND6A*, *PtrWND6B*, and *PtrMYB28*).

To test the binding regions in the promoters of *PtrC4H1, Ptr4CL5*, *PtrXTH5, PtrEXPA8*, *PtrGA20ox4, PtrGA3ox1, PtrWND6A*, *PtrWND6B*, and *PtrMYB28* by *PtrWOX13A in vivo*, chromatin immunoprecipitation (ChIP) assays were performed using transgenic poplars, which expressed Flag-tagged PtrWOX13A protein. PtrWOX13A–Flag-bound DNA fragments were immunoprecipitated using anti-Flag antibodies, and purified DNA fragments were used as templates in PCR analysis. The primer pair spanning DNA fragments of P1, P2, and P3 in *PtrC4H1*, P1 in *Ptr4CL5*, P1 and P2 in *PtrXTH5* and *PtrEXPA8*, P1 in *PtrGA20ox4*, *PtrGA3ox1*, *PtrWND6A, PtrWND6B* and P1, P2, P3, and P4 in *PtrMYB28* promoter sequences were designed (Figure 8A). The results showed that PtrWOX13A bound directly to the promoters of *PtrC4H1*, *Ptr4CL5*, *PtrXTH5*, *PtrEXPA8*, *PtrGA20ox4*, *PtrGA3ox1*, *PtrWND6A*, and *PtrMYB28* to regulate their expression (Figure 8B). These results, together with other pieces of evidence shown above, suggested that PtrWOX13A acted as a higher hierarchical regulator to directly activate downstream middle-layered TFs and structural genes involved in SCW component biosynthesis and GA homeostasis.

## Discussion

Multiple studies on herbaceous plants demonstrated that *WOX* genes participated in diverse development processes including shoot apical meristem (Nardmann and Werr, 2006), lateral organ development (Deveaux et al., 2008), plant stem cell maintenance (Sarkar et al., 2007; Stahl et al., 2009), and floral determinacy (Deveaux et al., 2008). In addition, although it has been reported that some members of the *WOX* ancient clade were involved in secondary growth (Romera-Branchat et al., 2013; Denis et al., 2017; He et al., 2019), none of the members of the woody plant WOX ancient clade gene family regulating wood formation has been reported. Therefore, *PtrWOX13A* is the first *WOX* ancient clade gene in tree that has been found to regulate SCW component biosynthesis.

### *PtrWOX13A* Regulated Secondary Cell Wall and Fiber Formation Through Regulating Transcription Factors and Structural Genes Related to Wood Formation

Overexpression of *PtrWOX13A* increased lignin and hemicellulose contents, wall thickness, lengths, and diameter of the fiber, and did not alter cellulose content (Figures 3, 5). These changes aligned well with the changes in TFs and structural genes involved in the SCW component biosynthesis (Zhong et al., 2010; Nakano et al., 2015; Gunasekara et al., 2016). As a result, significant changes in *PtrWOX13A* transgenic lines were conspicuous (Figure 6). In addition, the transient expression assay analysis revealed that the activities of the promoters of some TFs and structural genes, including *PtrC4H1*, *Ptr4CL5*, *PtrXTH5*, *PtrEXPA8*, *PtrWND6A*, and *PtrMYB28*, were directly activated by PtrWOX13A (Figures 7C, 8B). In summary, these data suggested that *PtrWOX13A* participated in wood formation as a higher hierarchy TF in the transcriptional regulation network of poplar SCW formation.

### *PtrWOX13A* Regulated Secondary Cell Wall and Fiber Formation Through Modulation of Gibberellin-Mediated Signaling Pathway

It has been recognized for a long time that bioactive GAs promoted SCW biosynthesis (Wang et al., 2013, 2017; Yuan et al., 2019), generally through promoting DELLA repressor degradation which leads to the activation of SCW master TFs (Biemelt et al., 2004). For example, GA is found to trigger the transactivation of SCW master switch NAC29/31, resulting in activating the expression of *MYB61*, which specifically upregulates *CESAs* and thereby enhances the cellulose biosynthesis in rice (Huang et al., 2015). Presumably, upregulation or downregulation of GA homeostasis key genes, such as *GA20ox*, *GA3ox*, and *GA2ox* (Yamaguchi, 2008), alters bioactive GA contents and affects SCW formation, especially lignin deposition, in the stems of several plants.

The previous studies have revealed that some *WOX* family members regulate plant development through modulating GA-homeostasis and GA-signaling. For example, WOX8 and WOX9 activities are associated with GA signaling in the internode elongation of *Arabidopsis* (Wang et al., 2014), while *WOX14* overexpression promotes the bioactive GA accumulation through activating *GA3ox* and repressing *GA2ox*, and thus enhances lignification of transgenic plants (Denis et al., 2017). Moreover, OsWOX3A participates in diverse developmental processes through negative feedback that controls the GA biosynthetic pathway (Cho et al., 2016). Similarly, *PtrWOX13A* overexpression resulted in the increased expression of *PtrGA20ox4* and *PtrGA3ox1* and decreased expression of *PtrGA2ox1* (Figure 6B), accompanied by the increased contents of bioactive GAs (Figure 5E). Thus, *PtrWOX13A* perhaps regulated wood formation partially through GA-mediated signaling cascade, such as DELLA-NAC (Huang et al., 2015).

### Possibility for *PtrWOX13A* to Modulate Bioactive Gibberellin Homeostasis Through Negative Feedback Regulation Pathway

In this study, we found that both *PtrGA20ox4* and *PtrGA3ox1* were significantly upregulated, while the expression of the *PtrGA2ox1* significantly decreased in *PtrWOX13A* overexpression lines (Figure 6B), which was also aligned well with the increased contents of bioactive GAs (Figure 5E). In addition, we showed that PtrWOX13A directly activated the promoter of *PtrGA3ox1* by binding to the TTAATSS *cis*-regulatory element in *PtrGA3ox1* and *PtrGA20ox4* promoter, but not the promoter of *PtrGA2ox1* (Figure 7C). This result resembles the previous report that *OsWOX3A* acts as both a positive regulator of auxin-related genes and a negative regulator of *YABBY3* in leaf development (Dai et al., 2007; Cho et al., 2013). Moreover, *PtrWOX13A* had a GA-responsive *cis*-regulatory element, P-BOX, in its promoter, and its expression rapidly increased within 24 h and then decreased after 24 h under exogenous GA treatment (Figure 1B), which indicates that the high bioactive GAs beyond a threshold might repress *PtrWOX13A.* Thus, when the activation of *PtrGA20ox4* and *PtrGA3ox1* and the repression of *PtrGA2ox1* resulting from *PtrWOX13A* overexpression caused the active GAs to increase beyond the threshold, the expression levels of *PtrWOX13A* decreased, resulting in the downregulation of the aforementioned genes regulating GA homeostasis, which then decreased the concentration of bioactive GAs. These results indicate that *PtrWOX13A* not only acts as a positive regulator and a negative regulator in GA homeostasis but also participates in GA homeostasis and signaling through the negative feedback regulation pathway in poplar. However, further studies are needed to test this.

### The Putative Regulatory Model of *PtrWOX13A* on Wood Formation

**FIGURE 9:**
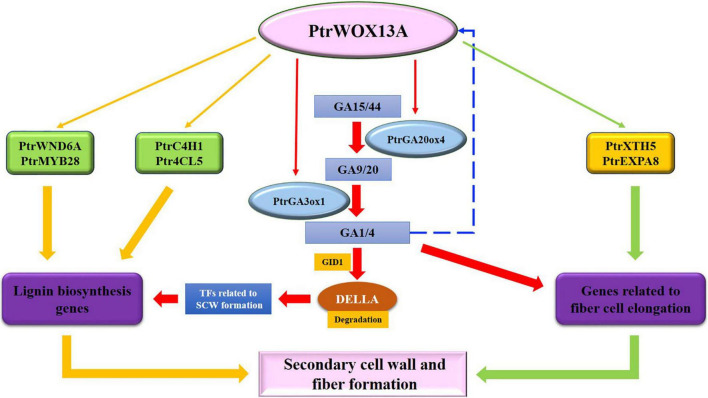
Postulated regulatory model of the *PtrWOX13A* regulatory network.

Finally, we proposed the two putative regulation pathways of *PtrWOX13A* participating in wood formation (Figure 9): (1) As a higher hierarchical switch TF, *PtrWOX13A* directly and indirectly regulated SCW TFs and structural genes; (2) *PtrWOX13A* enhanced bioactive GA contents through regulating the key genes regulating bioactive GA homeostasis and subsequently promoted DELLA proteins degradation through GA-mediated signaling cascade to derepressed wood-associated NAC or MYB TFs. The *PtrWOX13A* could participate in poplar wood formation through one or two of these pathways. This study revealed a new higher hierarchical regulator and its multiple regulatory chains in the hierarchical network governing SCW biosynthesis in tree species. The findings are instrumental for the molecular breeding of tree species for high biomass productivity.

## Data Availability Statement

The datasets presented in this study can be found in online repositories. The names of the repository/repositories and accession number(s) can be found in the article/Supplementary Material.

## Author Contributions

YZ and YL conducted most of the experiments and data analysis. XW and RW contributed to histochemical staining. XC and SW contributed to the vegetative propagation of *Populus trichocarpa* plantlets and phenotype analysis. HW contributed to figure preparation and manuscript revision. ZW designed the experiments and wrote the manuscript. All authors read and approved the final version of the manuscript.

## Conflict of Interest

The authors declare that the research was conducted in the absence of any commercial or financial relationships that could be construed as a potential conflict of interest.

## Publisher’s Note

All claims expressed in this article are solely those of the authors and do not necessarily represent those of their affiliated organizations, or those of the publisher, the editors and the reviewers. Any product that may be evaluated in this article, or claim that may be made by its manufacturer, is not guaranteed or endorsed by the publisher.

## Footnotes

1
http://www.ebi.ac.uk/Tools/msa/clustalw2/

2
https://phytozome-next.jgi.doe.gov/

3
https://meme-suite.org/meme/tools/meme

4
http://bioinformatics.psb.ugent.be/webtools/plantcare/html/

5
http://rsbweb.nih.gov/ij/

## Supplementary Material

Supplementary Figure 1 Phylogenetic analysis and protein sequence alignment of PtrWOX13. **(A)** Phylogenetic tree of WOX proteins from *Populus trichocarpa* and *Arabidopsis thaliana*. PtrWOX13 proteins were shown in an orange trapezoid. The tree showed three major phylogenetic subfamilies (subfamilies I–III) that are marked with different colors. **(B)** AtWOX13A (AT4G35550) protein of *A. thaliana* aligned with PtrWOX13 proteins from *Populus trichocarpa* (Potri.005G101800, Potri.002G008800, and Potri.005G252800). The conserved HD and MOG motifs represent by blue arrows and green arrows, respectively; yellow, red, brown, and black boxes represent the YxDpl motif, NVYNWFQNR motif, activation region, and ESExE motif, respectively.

Supplementary Figure 2 *Cis*-regulatory elements in promoter regions of *PtrWOX13* genes and motifs of PtrWOX13 proteins. **(A)** Motifs of PtrWOX13 proteins. Colors of boxes denote different motifs while lengths of boxes represent relative motif lengths. **(B)** Venn diagram of common and unique motifs present in promoter regions of *PtrWOX13A, PtrWOX13B1*, and *PtrWOX13B2*. **(C)** *Cis*-regulatory elements in the 2,000 bp promoter sequences upstream of transcription start codons of *PtrWOX13A*, *PtrWOX13B1*, and *PtrWOX13B2* genes using the online tool PlantCARE. These elements have different functions, which are represented by different colors.

Supplementary Figure 3 Identification of *PtrWOX13A* in *Populus trichocarpa* overexpression transgenic lines. **(A)** PCR detection of *PtrWOX13A* transgenic lines. Lane M, DNA marker DL2000; Lane 1, wild type; Lane 2–9. PCR products of *PtrWOX13A* transgenic lines. **(B)** qRT-PCR detection of *PtrWOX13A* transgenic lines. *PtrActin* was used as a control. Each error bar represents the standard deviation of three biological replicates. Asterisks represent levels of significance (Dunnett’s test; **p* < 0.01 < *p* < 0.05, ***p* < 0.01). **(C)** 90-day-old wild type (WT) and *PtrWOX13A* transgenic lines (OE-2, OE-3, and OE-7).

Supplementary Figure 4 Anatomical and histochemical analyses of stems of different developmental stages in *Populus trichocarpa* transgenic lines. **(A–D)** Represent phloroglucinol-HCl stained transverse sections of the internode 2 (IN2), 4 (IN4), 6 (IN6), and 8 (IN8), respectively. **(E–H)** Represent toluidine blue O-stained transverse sections of IN2, IN4, IN6, and IN8 in WT, respectively. **(I–L)** were phloroglucinol-HCl-stained transverse sections of IN2, IN4, IN6, and IN8 in *PtrWOX13A* transgenic lines, respectively. **(M–P)** Represent toluidine blue O-stained transverse sections of IN2, IN4, IN6, and IN8 in *PtrWOX13A* transgenic lines, respectively. Scale bars = 200 μm. The toluidine blue and phloroglucinol-HCl were used to stain cell morphology and lignin, respectively.

Supplementary Figure 5 Analysis of PtrWOX13A binding to *cis*-acting elements by yeast one-hybrid assay. The pHIS2-p53/pGADT7-rec2-p53 and pHIS2-p53/pGADT7-rec2-PtrWOX13A were used as the positive and negative control, respectively. A represents ATTGATTG *cis*-regulatory element; AM1 and AM2 represent mutated ATTGATTG *cis*-regulatory elements; T represents TTAATSS *cis*-regulatory element; TM1 and TM2 represent mutated TTAATSS *cis*-regulatory elements; 10^–1^, 10^–2^, and 10^–3^ represent solution dilution ratio of transformed Y187 yeast cells.
