# Supplementary figures and images for: PtrWOX13A Promotes Wood Formation and Bioactive Gibberellins Biosynthesis in Populus trichocarpa
Source: Front Plant Sci. 2022 Jun 28;13:835035. doi: 10.3389/fpls.2022.835035 (PMC9274204; doi:10.3389/fpls.2022.835035)

A

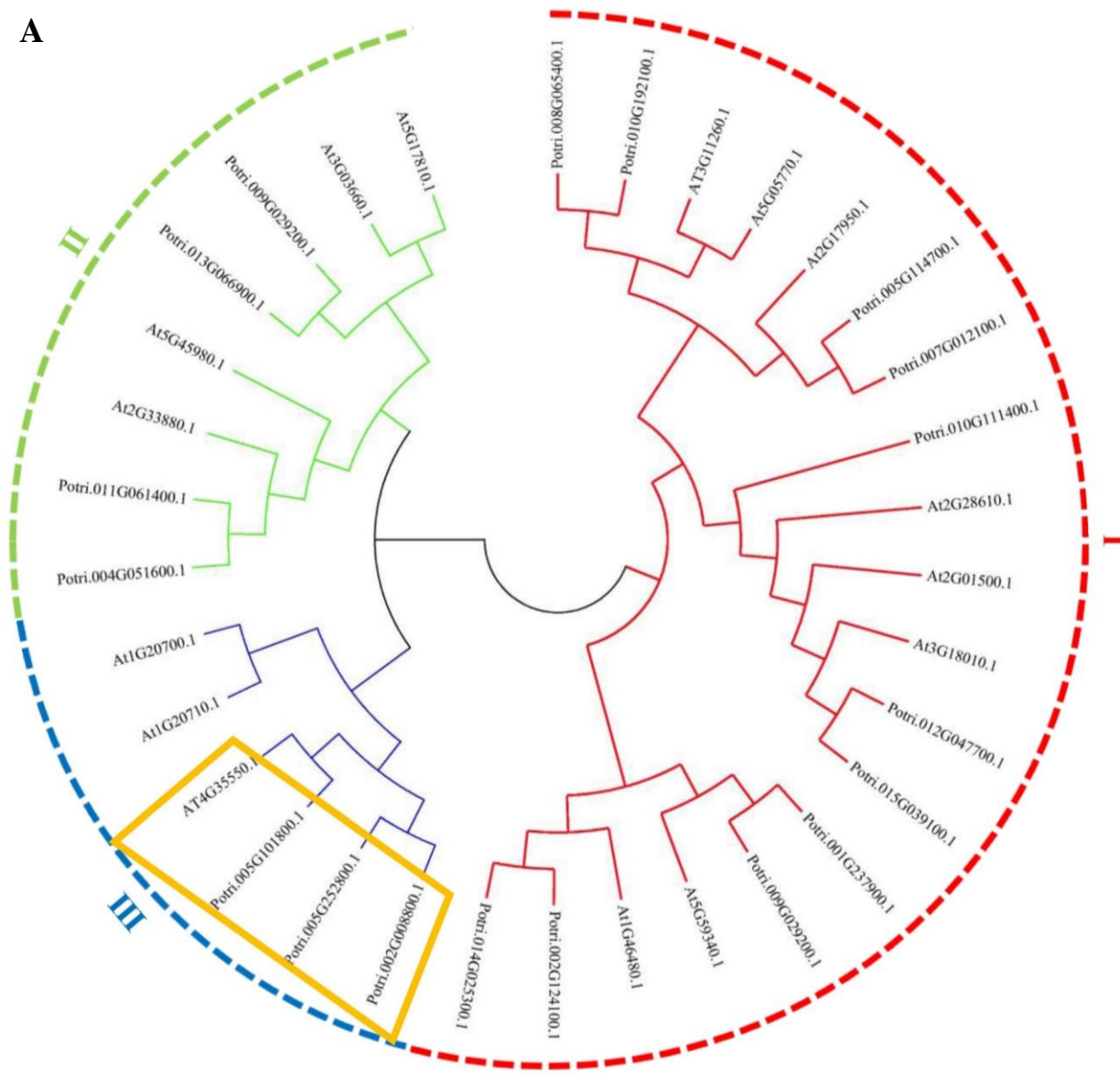

B

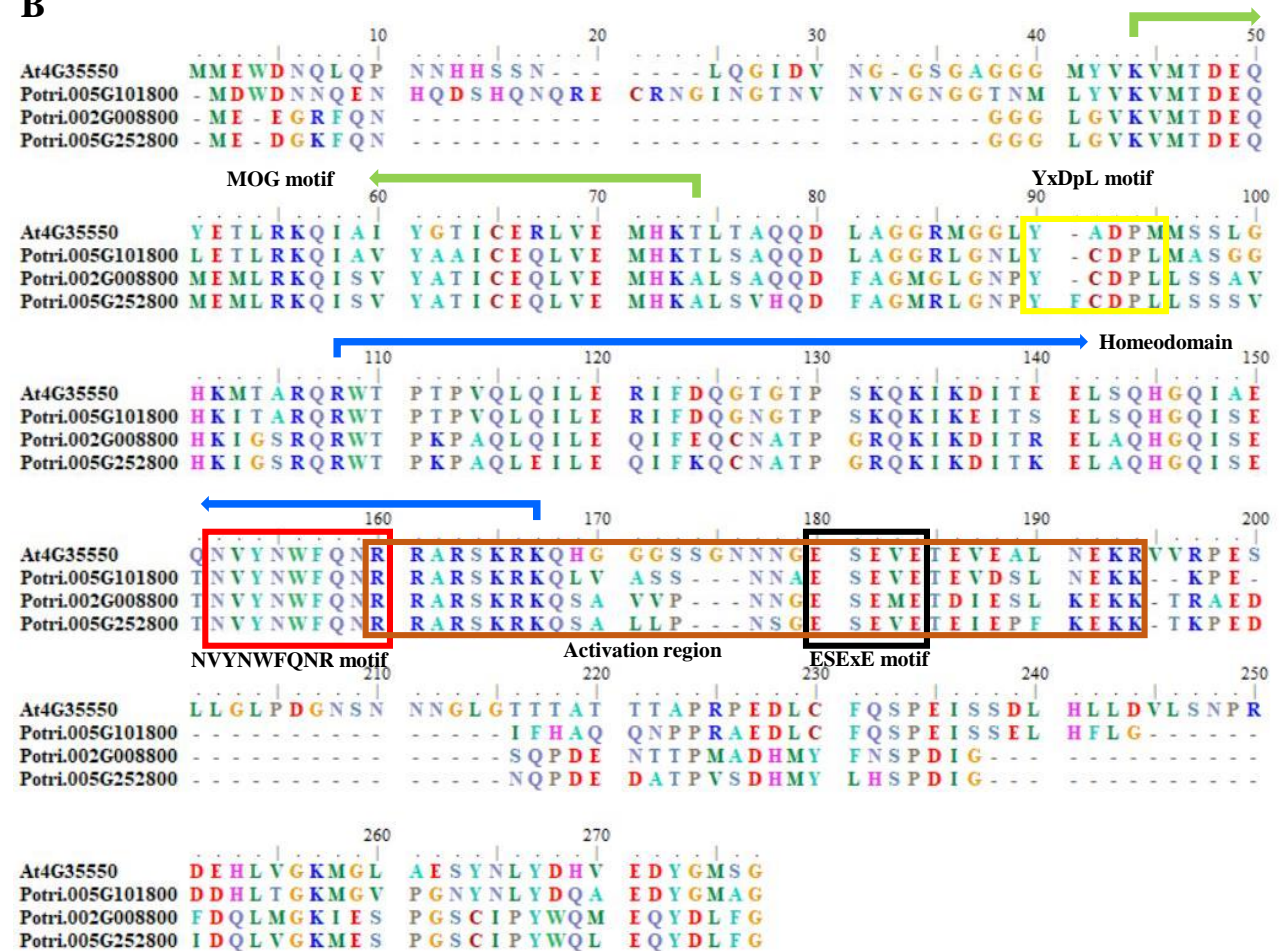

A

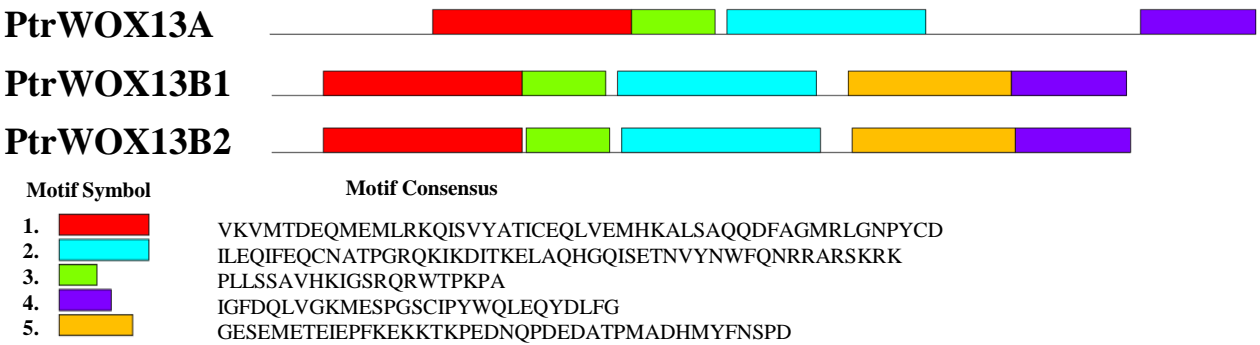

B

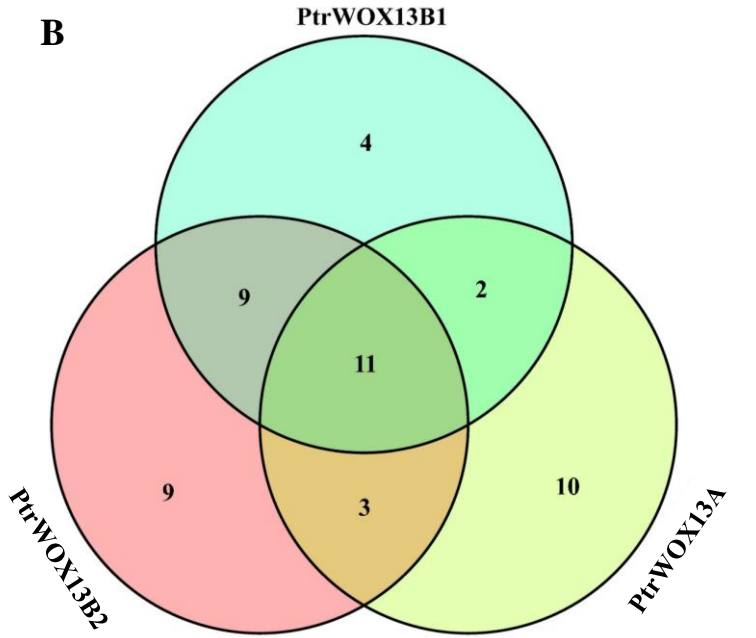

C

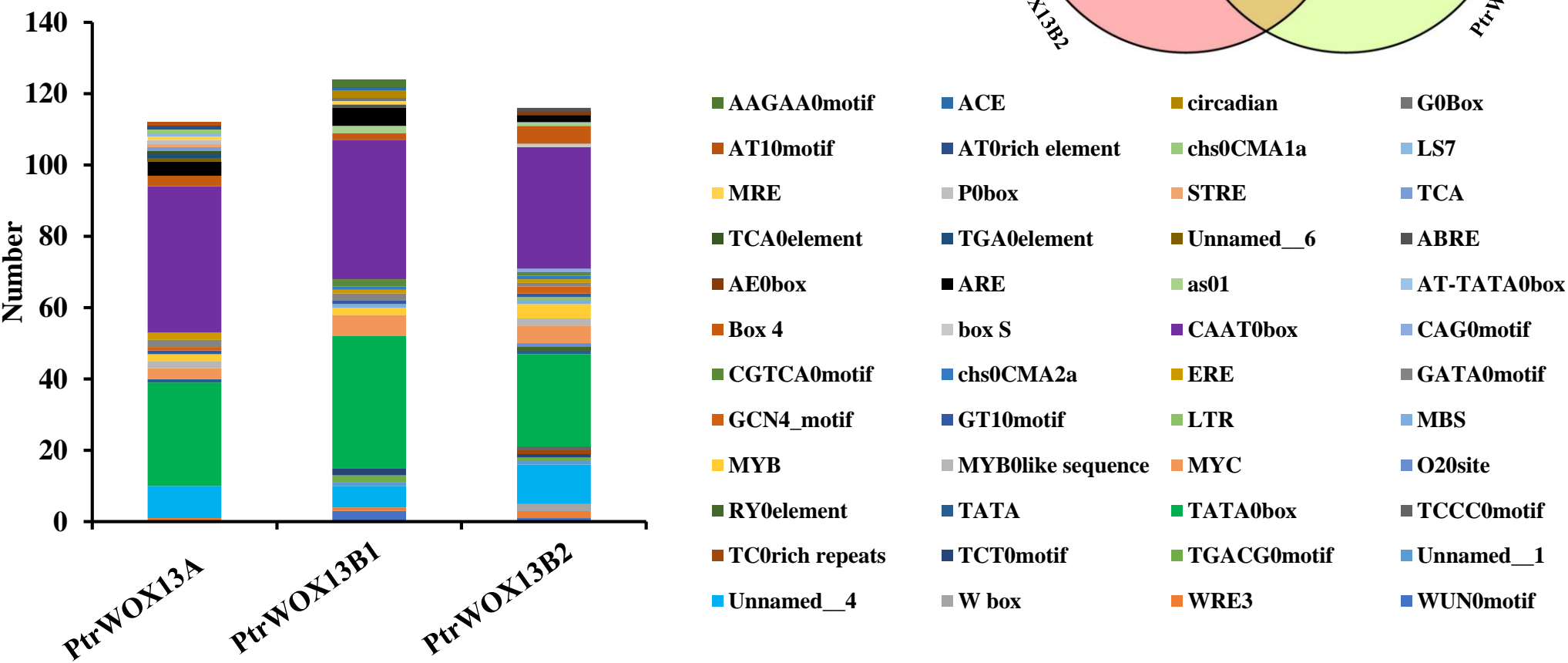

**A**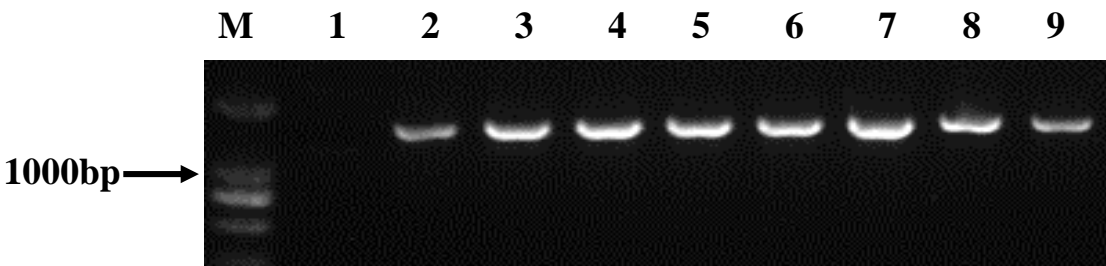**B**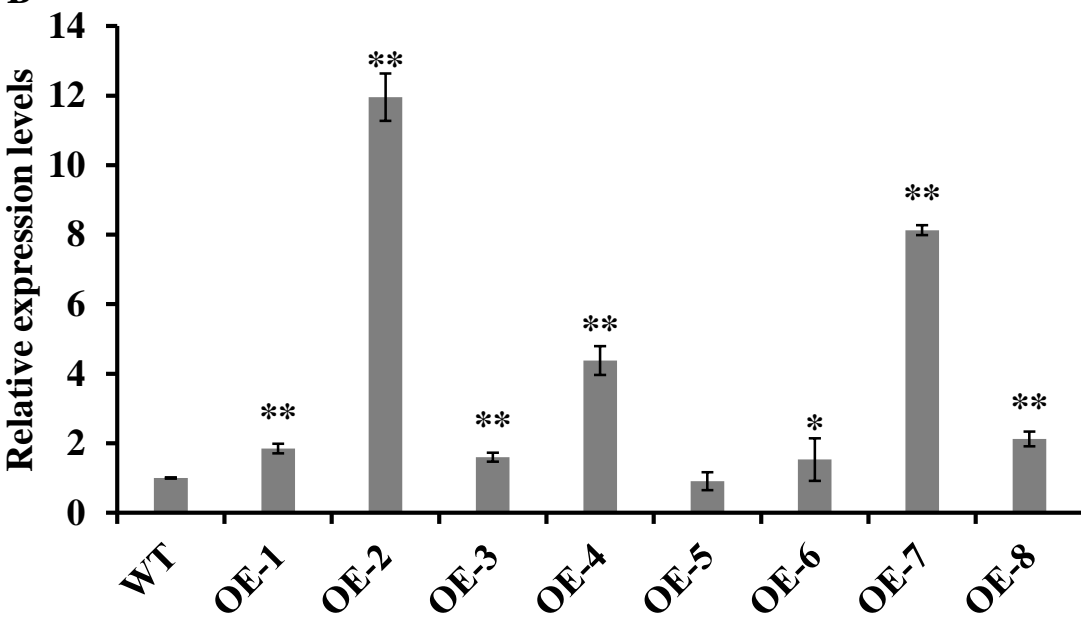**C**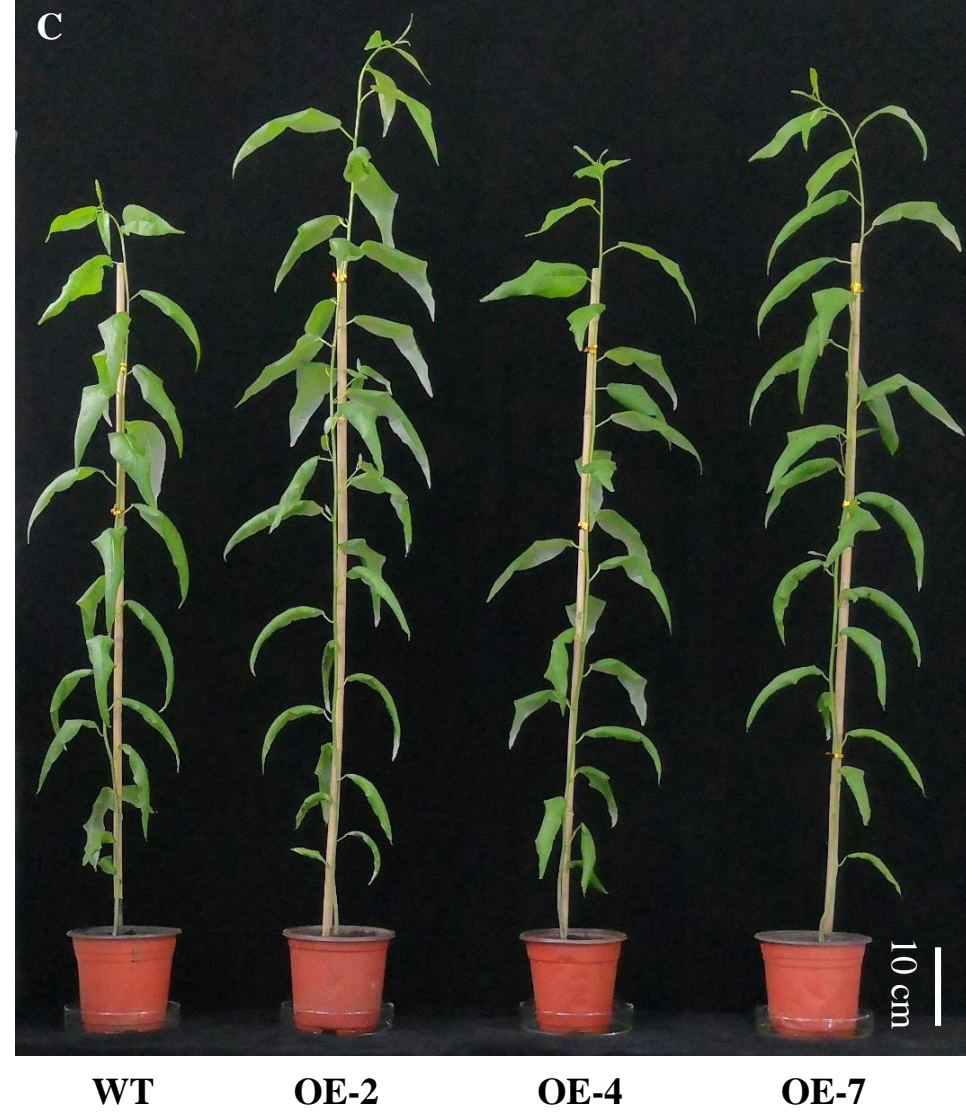

**WT*****PtrWOX13A*-OE****IN2**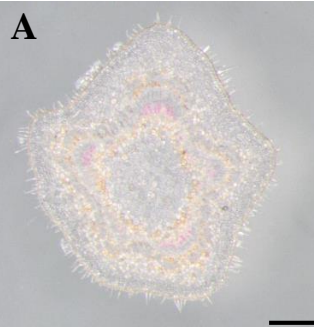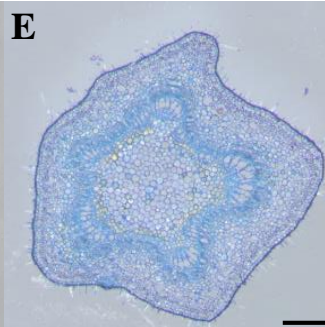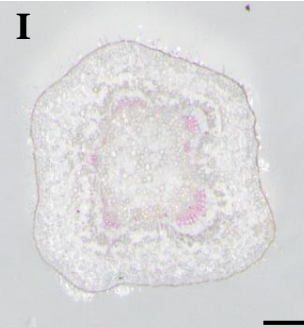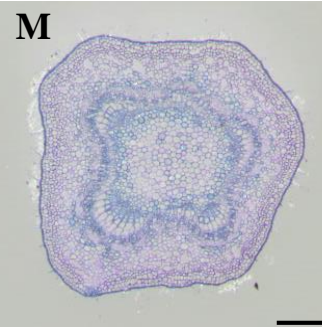**IN4**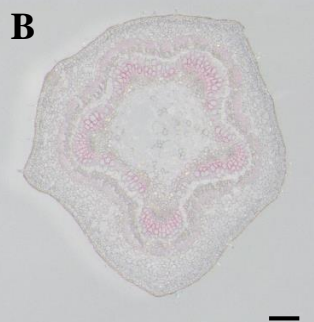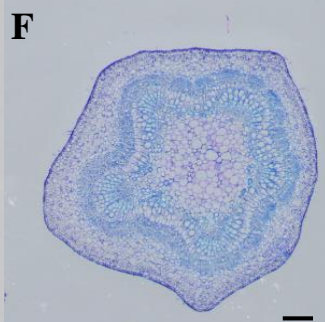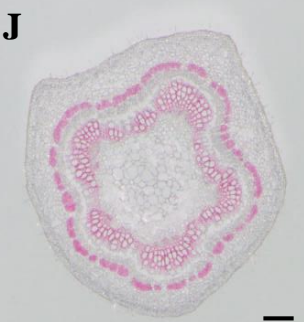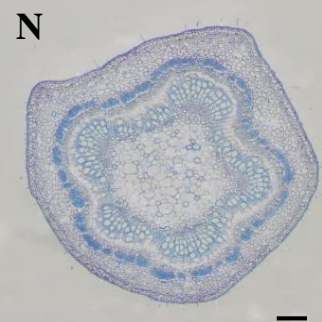**IN6**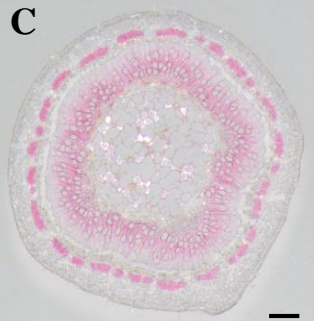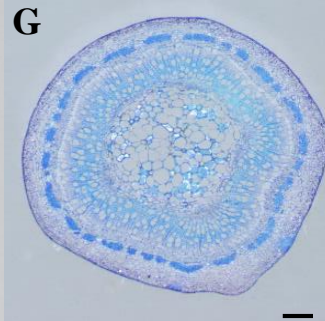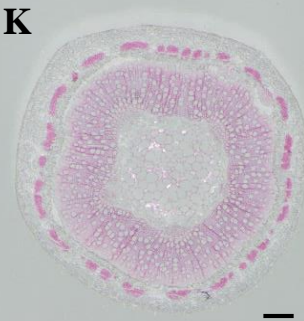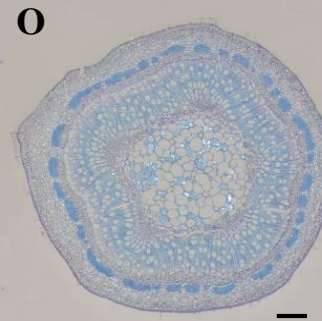**IN8**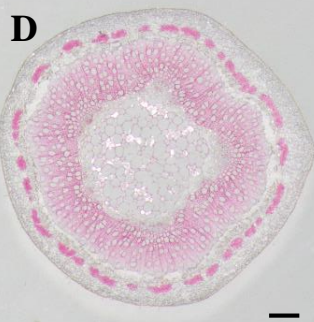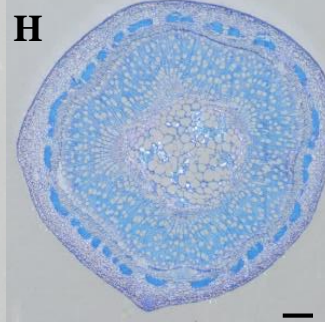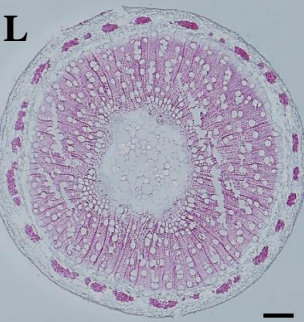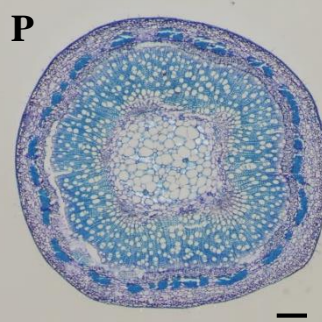

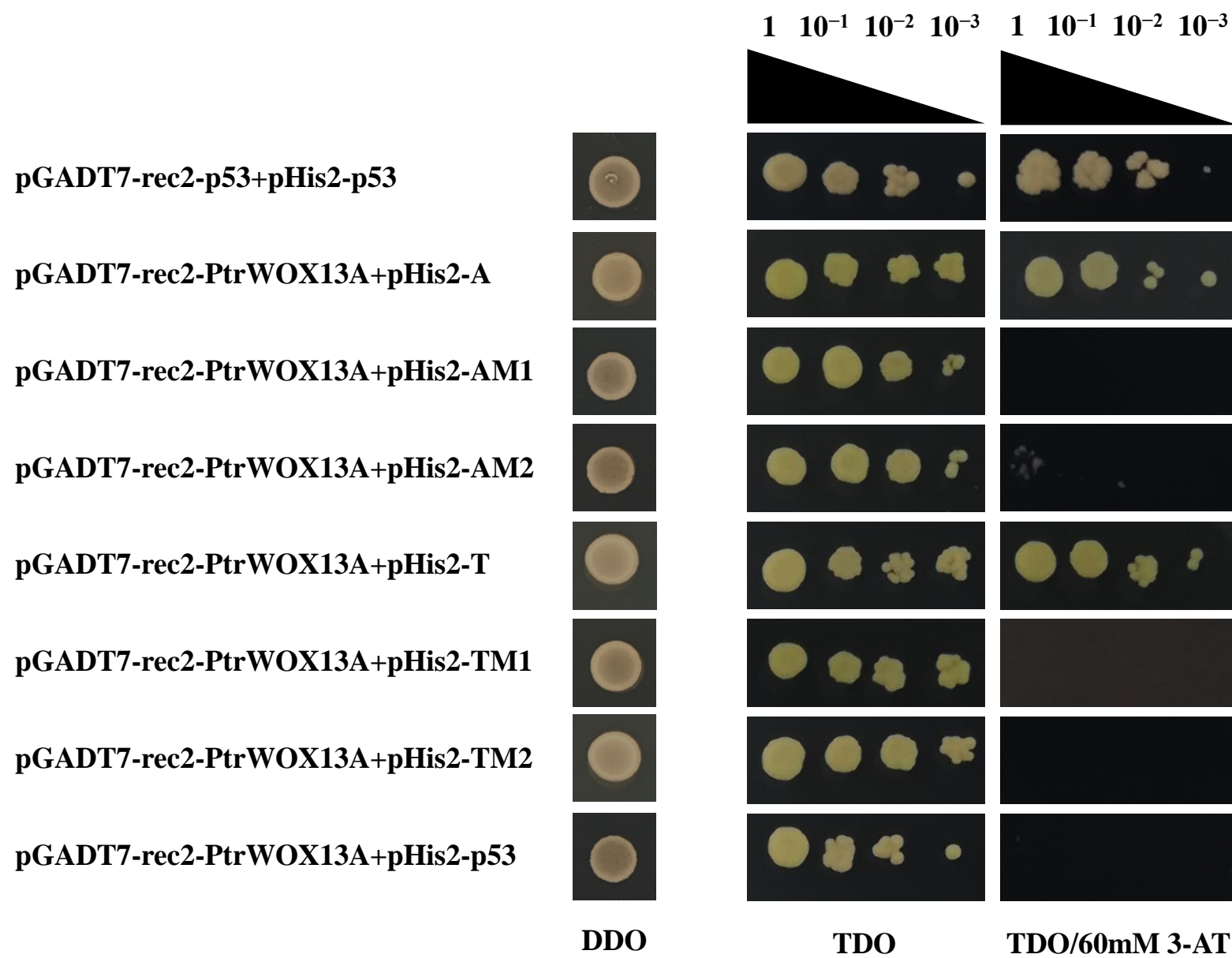

Supplement: Supplementary file 1 [file Data_Sheet_1.zip › Additional files/Additional files Figures.pdf]
